# Role of ultrasound in the diagnosis of cervical tuberculous lymphadenitis in children

**DOI:** 10.1007/s12519-021-00453-w

**Published:** 2021-09-01

**Authors:** Tian-Zhuo Yu, Ying Zhang, Wen-Zhi Zhang, Gao‑Yi Yang

**Affiliations:** Department of Ultrasonography, Affiliated Hangzhou Chest Hospital, Zhejiang University School of Medicine, Chinese and Western Hospital of Zhejiang Province (Hangzhou Red Cross Hospital), No. 208 Huancheng East Road, Hangzhou, 310003 China

**Keywords:** Cervical lymphadenitis, Children, Tuberculosis, Ultrasonography

## Abstract

**Background:**

To describe sonographic characteristics of cervical tuberculous lymphadenitis (CTBL) in children, clinical information, and sonograms of 348 lymph nodes (LNs) from 57 children with CTBL were retrospectively analyzed in this study.

**Methods:**

We retrospectively reviewed the clinical data and sonograms of 348 LNs from 31 boys and 26 girls with CTBL, who were confirmed by pathology or laboratory examination, at the Hangzhou Red Cross Hospital between June 2014 and December 2020. The age of the children ranged from 1 to 14 years (average 7.1 ± 2.9 years).

**Results:**

Night sweats, fatigue and loss of appetite were the most common clinical symptoms observed in children with CTBL. Unilateral LN involvements were common. Occasionally, CTBL was found in healthy children with no symptoms. On sonography, the hilus was absent or unclear in all LNs. The short-to-long axis (S/L) ratio was ≥ 0.5, and the edges were unclear in most LNs. Other accompanying findings included necrosis (47.4%), an echogenic thin layer (36.8%), surrounding soft-tissue edema (38.5%), multiple intra-nodal strong echo (28.2%), sinus (22.7%) and abscess formation (6.9%). The Doppler ultrasound showed that the majority of vascularity patterns of CTBL were capsular or peripheral (33.3%).

**Conclusions:**

Ultrasound is a recommended examination method for children from different age groups with cervical lymphadenitis. The ultrasonic signs of hilus absence, S/L ratio ≥ 0.5, unclear edge, necrosis, echogenic thin layer, strong echoes and capsular or peripheral vascularity may aid in the diagnosis of cervical tuberculous lymphadenitis.

## Introduction

According to the World Health Organization, an estimated 10.0 million (range 8.9–11.0 million) people were diagnosed with tuberculosis (TB) worldwide in 2019, and children (aged < 15 years) accounted for 12% of this population [1]. Early diagnosis and treatment of TB in children is a key factor in reducing adult TB and is an important link in controlling transmission. Although diagnosis of TB in children is challenging and less studied, radiological findings may provide useful information for diagnosis [2–4].

Tuberculous lymphadenitis is the most common form of extrapulmonary TB in childhood and is estimated to comprise up to 24% of all TB cases in children. Among tuberculous lymphadenitis cases, 60–90% are cervical tuberculous lymphadenitis (CTBL) [5, 6]. Conventional ultrasound has the advantages of high resolution, real-time evaluation, relatively low cost and no radiation. As a recommended imaging method for the examination of cervical lymph node (LN) diseases, conventional ultrasound is more suitable for children with CTBL [7, 8].

In this study, ultrasonographic images of 348 LNs from 57 children with CTBL were analyzed to evaluate the application value of ultrasound in the diagnosis and treatment of CTBL.

## Methods

We retrospectively reviewed the ultrasound images of 646 cervical LNs in 196 patients at our hospital between June 2014 and December 2020. All cases had been diagnosed by clinicians through pathology, laboratory examination or diagnostic therapy, and we obtained the diagnosis results from the medical record system. If the child had several ultrasonography, we only studied the images before the start of treatment to ensure the accuracy of the images. This study was approved by the Human Subject Ethics Subcommittee of the authors’ institution. The study procedures conformed to the provisions of the Helsinki Declaration.

All ultrasonographic explorations were performed on an iU22 system (Philips, Amsterdam, The Netherlands) with a L12-5 linear probe and a LOGIQ E9 general imaging system (GE Healthcare, Chicago, IL, USA) with a high‑frequency probe (6‑15 MHz). The children were kept in the supine position with their head dropped back. Suspicious LNs were searched according to the corresponding anatomical regions [9].

The imaging features included numbers, size, shape, echogenic hilus, edge, internal echogenicity, calcification, necrosis, matting and adjacent soft tissue edema. The shape of the LNs was determined by the short-to-long axis (S/L) ratio. Strong echo is defined as hyperechoic similar to bone or calcification.

The vascular pattern of LNs was evaluated with color or power Doppler ultrasound, while the blood flow velocity and vascular resistance were also measured. Vascularity patterns of LNs were divided into (1) hilar: flow signals branching radially from the hilum; (2) capsular or peripheral: flow signals along the periphery of the LNs; (3) mixed: presence of hilar and capsular flow and (4) avascular: no flow signal [10].

Normally distributed data were presented as mean ± standard deviation. The data between groups were analyzed by the *χ*^2^ exact test. Descriptive statistics were used to characterize the patients and the sonographic data. All statistical analyses were performed by the Statistical Package for the Social Sciences (SPSS) software (version 20, IBM Corporation, Armonk, NY, USA). A *P* value < 0.05 indicated statistical significance.

## Results

Of these 646 LNs, 348 (53.9%) from 57 children were tuberculous lymphadenitis and 298 (46.1%) from 139 children were other lymphadenectasis. Among them, 113 LNs were reactive hyperplasia, 98 LNs were nonspecific acute and chronic lymphadenitis, 62 LNs were Kikuchi disease, 12 LNs were infectious mononucleosis, 6 LNs were cat-scratch disease, 4 LNs were lymphoma, 2 LNs were *Brucella* infection and only one was metastatic. Sex, age and number of children and lymph nodes according to the final diagnosis are shown in Table 1. There was no statistical difference in age and sex compared between the two groups. Comparison of sonographic findings of tuberculous lymphadenitis and other lymphadenectasis are shown in Table 2. Due to the unbalanced nature of the sample size, we did not compare the differences between tuberculous lymphadenitis and lymphomas and provided only initial data to avoid conclusion bias.

**Table 1:** Sex, age and number of children and lymph nodes

Characteristics	Tuberculosis	Other	*P*
No. of children/lymph node	57/348	139/298	
Age (y), mean ± SD	7.1 ± 2.9	6.9 ± 3.1	0.346
Sex, *n* (%)			
Boy	31 (54.4)	64 (46.0)	0.289
Girl	26 (45.6)	75 (54.0)	

*SD* standard deviation

**Table 2:** Comparison of sonographic findings of tuberculous lymphadenitis and others

Sonographic findings	Number of lymph nodes					*P*^b^	*P*^c^
	Tuberculosis (*n* = 348)		Reactive lymphadenitis (*n* = 113)	Lymphoma (*n* = 4)	Other lymphadenopathy^a^ (*n* = 181)		
S/L						< 0.001	< 0.001
< 0.5	110 (31.6)		95 (84.1)	0 (0)	144 (79.6)		
≥ 0.5	238 (68.4)		18 (15.9)	4 (100)	37 (20.4)		
Edge						< 0.001	< 0.001
Clear	146 (42.0)		107 (94.7)	3 (75.0)	163 (90.1)		
Not clear	202 (58.0)		6 (5.3)	1 (25.0)	18 (9.9)		
Strong echo	98 (28.2)		0 (0)	0 (0)	2 (1.1)	< 0.001	< 0.001
Hilus absent or unclear	348 (100)		2 (1.8)	4 (100)	22 (12.2)	< 0.001	< 0.001
Necrosis	165 (47.4)		1 (0.9)	0 (0)	30 (16.6)	< 0.001	< 0.001
An echogenic thin layer	128 (36.8)		0 (0)	0 (0)	5 (2.8)	< 0.001	< 0.001
Surrounding soft-tissue edema	134 (38.5)		38 (33.6)	1 (25.0)	87 (48.1)	0.352	0.034
Sinus	79 (22.7)		0 (0)	0 (0)	2 (1.1)	< 0.001	< 0.001
Abscess formation	24 (6.9)		0 (0)	0 (0)	6 (3.3)	< 0.001	0.091
Vascularity patterns						< 0.001	< 0.001
Capsular or peripheral	116 (33.3)		3 (2.6)	0 (0)	20 (11.1)		
Avascular	74 (21.3)		2 (1.8)	0 (0)	50 (27.6)		
Hilar	68 (19.5)		106 (93.8)	3 (75.0)	79 (43.6)		
Mixed	90 (25.9)		2 (1.8)	1 (25.0)	32 (17.7)		

Values are *n* (%). *S/L* short-to-long axis ratio, *LN* lymph nodes. ^a^98 LNs were nonspecific acute and chronic lymphadenitis, 62 LNs were Kikuchi disease, 12 LNs were infectious mononucleosis, 6 LNs were cat-scratch disease, 2 LNs were *Brucella* infection and one LN was metastatic; ^b^tuberculosis vs. reactive lymphadenitis; ^c^tuberculosis vs. other lymphadenopathy

The clinical data and ultrasound images of children with CTBL were analyzed as follows: the age of the patients ranged from 1 to 14 years (average 7.1 ± 2.9 years). A single child may have had more than one symptom. Sixteen children had no systemic symptoms and were in good health. Lymphadenopathy was accidentally found in these children. All cases had subjected to core needle biopsy of the LN, and then the material was subjected to histopathology examination, acid fast bacilli (AFB) staining, gene-Xpert and BATEC MGIT 960. When any of these methods is positive, the clinical diagnosis of tuberculous lymphadenitis is clear. The positivity rate on gene-Xpert *Mycobacterium tuberculosis bacilli* (MTB) was 75.4% (43/57), 30 cases were diagnosed by histopathology, 19 cases were diagnosed by presence of AFB on smear, BATEC MGIT 960 system was used to confirm a diagnosis in 18 cases. We obtained the diagnosis results from the electronic medical record system. Chest radiographs of 57 children showed radiographic signs of pulmonary infection in 12 cases. Neck computed tomography (CT) was performed on only eight of the children, which revealed multiple cervical lymphadenopathy. Of them, images of three children showed calcification. Two children had lymphadenopathy in other anatomical regions, and one child had tuberculosis of pericardium. All the children had been vaccinated with Bacille Calmette-Guerin. Only 11 children were tested for human immunodeficiency virus, and the results were negative. They were divided into infant groups (1–3 years), preschoolers (3–7 years) and children's groups (7–14 years) based on the age of the child. The clinical information of 57 children with CTBL are shown in Table 3.

**Table 3:** Clinical information of 57 children with cervical tuberculous lymphadenitis

Clinical information	Age (y)			Total (*n* = 57)
	1 to < 3	3 to < 7	7–14	
Boy	10	13	8	31 (54.4)
Girl	9	11	6	26 (45.6)
Unilateral	14	21	5	40 (70.2)
Bilateral	5	3	9	17 (29.8)
Night sweats	16	15	8	39 (68.4)
Fatigue^a^	4	13	10	27 (47.4)
Loss of appetite	3	9	7	19 (33.3)
Weight loss^b^	2	3	7	12 (21.0)
Fever	4	3	1	8 (14.0)
Cough	1	3	3	7 (12.3)
No symptoms	3	9	4	16 (28.1)
Chest radiographs positive^c^	2	5	5	12 (21.0)
Household contacts of people with TB	7	5	1	13 (22.8)
TB in other anatomical regions	2	7	6	15 (26.3)
Gene-Xpert MTB positive	11	20	12	43 (75.4)
Histopathology positive^d^	8	14	8	30 (52.6)
AFB positive	6	9	4	19 (33.3)
BATEC MGIT 960 positive	5	8	5	18 (31.6)

*MTB Mycobacterium tuberculosis*, *AFB* acid fast bacilli. ^a^Clinical data of 1 to < 3 years group and 3 to < 7 years group have documented somnolence and mental insufficiency in the medical record system, which we classify as fatigue; ^b^clinical data of 1 to < 3 years group have documented low body weight, which we classify as weight loss; ^c^radiographs positive expression was radiographic signs of pulmonary infection; ^d^histopathology positive expression was that the pathologic results showed tuberculous granuloma with caseous necrosis and suggested the diagnosis of tuberculosis

The mean number of LNs was 6.9 ± 2.5 (range 3–12), there was no difference between the age groups included in the study. The mean largest diameter (L) of the diseased LNs was 28.8 ± 7.2 mm (range 11–46 mm). The mean short diameter (S) of the diseased LNs was 15.8 ± 6.5 mm (range 7–30 mm). The hilus was absent or unclear in all LNs (348/348, 100%).

**Fig. 1:**
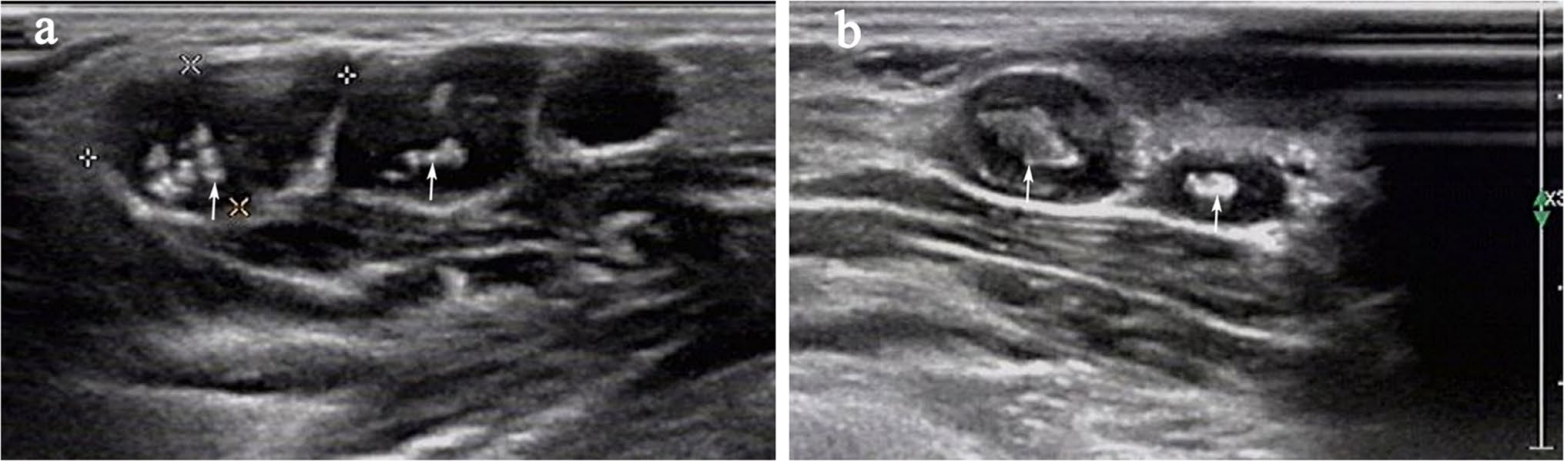
Cervical ultrasonography of the lymph nodes (LNs) shows strong echo. **a** Multiple strong echoes were seen within two of three lymph nodes lined up (arrows); **b** strong echoes were seen within two enlarged LNs (arrows)

**Fig. 2:**
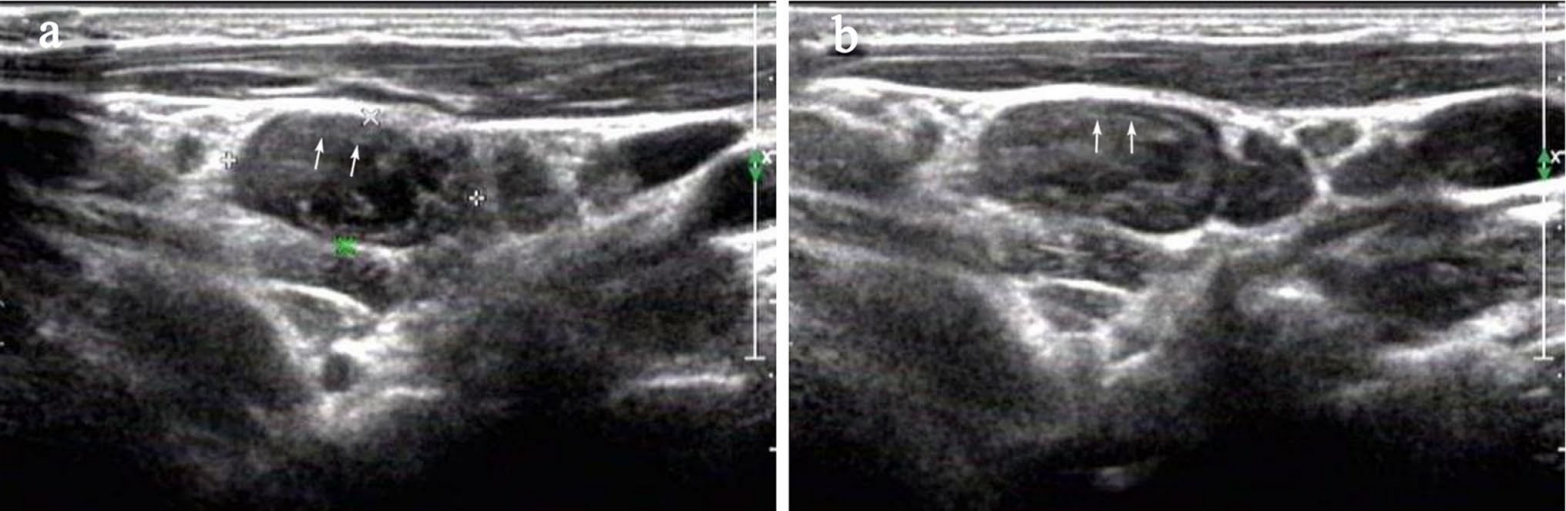
Cervical ultrasonography of lymph nodes (LNs) in a boy with tuberculous lymphadenitis. **a** Sonograms of LNs showing an echogenic thin layer with an irregular margin within the node, deep to the peripheral margin (arrows)**; b** an echogenic thin layer in the peripheral margin within another node is shown (arrows)

**Fig. 3:**
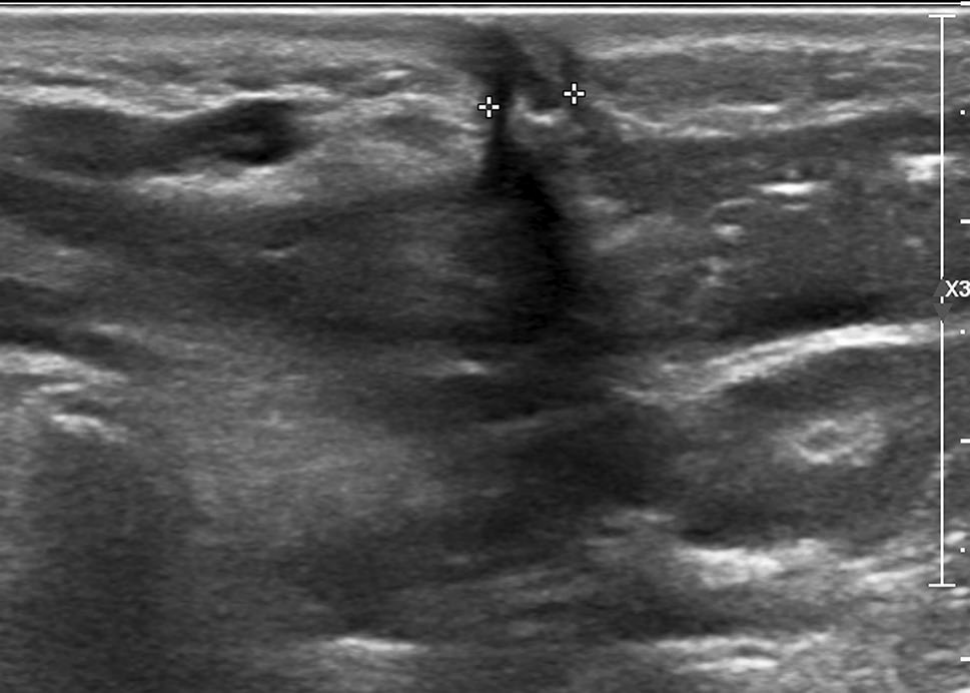
Cervical ultrasonography of lymph nodes shows abscess and sinus. Subcutaneous abscess is formed near the lymph nodes with ill-defined boundaries, and strips of hypoechoic sinuses are seen above extending into the skin

Sonography showed multiple intra-nodal strong echo in 98 (28.2%) LNs (Fig. 1). An echogenic thin layer was seen within 128 (36.8%) LNs (Fig. 2). The sinus tract was an irregular strip of hypoechoic tissue connecting LNs or abscesses to the skin (Fig. 3). The Doppler ultrasound showed that the vascularity patterns of LNs were capsular or peripheral (116/348, 33.3%), mixed (90/348, 25.9%), avascular (74/348, 21.3%) and hilar (68/348, 19.5%). Table 4 reports the sonographic findings of different age groups. There was no statistically significant difference in sonographic features among different age groups.

**Table 4:** Comparison of sonographic findings of different age groups

Sonographic findings	Percentage	Number of LNs of different age groups (y)			*x*^2^	*P*
		1 to < 3 (*n* = 133)	3 to < 7 (*n* = 126)	7 to 14 (*n* = 89)		
S/L ≥ 0.5	68.4 (238/348)	88	86	64	0.816	0.665
Edge not clear	55.2 (192/348)	75	71	46	0.588	0.745
Strong echo	28.2 (98/348)	35	37	26	0.363	0.834
Necrosis	47.4 (165/348)	66	57	42	0.502	0.778
An echogenic thin layer	36.8 (128/348)	51	45	32	0.228	0.892
Surrounding soft-tissue edema	38.5 (134/348)	50	48	36	0.198	0.906
Sinus	22.7 (79/348)	28	30	21	0.335	0.846
Abscess formation	6.9 (24/348)	9	9	6	0.019	0.991
Capsular or peripheral	33.3 (116/348)	42	43	31	0.310	0.856
Avascular	21.3 (74/348)	29	28	17	0.341	0.843
Hilar	19.5 (68/348)	28	23	17	0.337	0.845
Mixed	25.9 (90/348)	33	33	24	0.140	0.932

*LNs* lymph nodes, *S/L* short-to-long axis ratio

## Discussion

Lymphadenopathy may be caused by multiple infections, among which tuberculosis is one of the common causes of cervical LN enlargement in children [11]. Bacterial loads in LNs serve as important sites for persistence of significant numbers of MTB, which become the reservoir of future disease in adulthood [12, 13]. Ultrasound, which is the preferred tool for LN display and characterization of its size, shape, borders, internal architecture, vascularity, and perinodal soft tissues, plays an important role in the differential diagnosis of lymphadenopathy in children [14].

Tuberculous lymphadenitis usually presents as a slowly progressive, painless swelling of a single LN. Owing to the marginal symptom in the early stage, children with CTBL may largely be ignored by parents or doctors [12, 15]. A few weeks to months later, more LNs are involved, larger masses can be formed by further matting, and the common clinical picture comprises unilateral or bilateral multiple painless masses, which may be painful after secondary bacterial infection. The formation of suppurative nodes and intranodal abscesses may occur after caseation and liquefactive necrosis. Fistulization may form when the capsule of LNs is ruptured. Systemic symptoms, such as low-grade fever, weight loss, fatigue and night sweats may be present [5].

Baatenburg de Jong et al. [16] reported the ultrasound patterns of LN involvement according to different stages of the disease. Ultrasound images of LNs often show a non-specific hypoechoic pattern in an early granulomatous stage and a hypoechogenic mass with an inhomogeneous aspect in the caseation stage. As the disease progresses, abscess and sinus may be formed. These ultrasound patterns also were observed in the children included in this study. Therefore, it may be assumed that the pathological processes of MTB in children's LNs are similar to those in adults. The ultrasonographic findings of different lymph nodes in the same individual are not necessarily the same but were diversified in our study. In our study, all CTBL cases showed the absence of hilus and 68.4% of LNs showed S/L ratio ≥ 0.5, perhaps because granulomatosis produces deep morphological and structural alterations [17, 18]. The sign of strong echoes in the mass was thought to be characteristic of tuberculosis, which was present in approximately 33.3% of the cases in the study conducted by Asai et al. [19]. In our study, multiple intra-nodal strong echoes were observed on the sonogram in 28.2% of the LNs. There are studies that show ultrasound and CT showed the same rate of calcification in CTBL and ultrasound may even be equaled histopathology [16, 20]. However we found that one case, who showed intra-nodal strong echoes on ultrasound, did not show calcification in the nodule on CT but only showed hypoechogenicity that could not be distinguished from lymph node tissue. Moreover, the LN that was resected had no calcification but only had large areas of caseous necrosis in histopathology. Therefore, we believe that strong echoes on ultrasound may not only include the same manifestation as the calcification shown by pathology but also the manifestation of caseous necrosis, eventually resulting in healing [21]. Ultrasound may show caseous necrosis in CTBL more frequently than CT and is suitable for children during the treatment period.

Another important sign of CTBL in adults is an echogenic thin layer (2–3 mm) within the LN, deep to the peripheral margin, accounting for 86% in the study conducted by Asai et al. [19]. However, only 36.8% of the LNs showed this characteristic in our study. The possible reason was differences between the ability of LNs in children and adults to generate an immune response to *Mycobacterium tuberculosis*. Granulomas that form in LNs disrupt the LN structure by pushing out T cells and B cells, and destroy the normal vasculature. Epithelioid granulomas are encapsulated and progress to central caseous necrosis [13, 21]. Children may have fewer LNs involved in this process.

In addition, surrounding soft tissue edema appears as ill-defined echo-lucent areas in the peri-nodal soft tissues on ultrasound, with or without sinus and abscess formation in CTBL, particularly in the later course of the disease [22]. Several studies have reported that 43–74% of CTBL had surrounding soft tissue edema, which is related to periadenitis [23, 24]. This finding was seen in 38.5% of LNs of children in our study, and this percentage was not statistically significant in comparison with other lymphadenectasis. Our study found lower rates perhaps because children may suffer from less periadenitis caused by destruction of the LN capsule due to their lower immune function.

According to previous studies, sinus and abscess formation were uncommon in CTBL of adults, and the incidence of sinus was about 10% [15, 25]. However, in our study 22.7% and 6.9% of CTBL cases showed sinus and abscess, respectively. The higher rates were probably because children have thinner skin and subcutaneous soft tissue, and sinus tract may form more easily than in adults. When spread of nodal masses into the subcutaneous tissue or sinus are found in children with cervical lymphadenopathy, the possibility of tuberculosis should be considered first.

Peripheral angiogenesis can occur in tuberculous lymphadenitis in which persistent inflammatory stimuli and internal necrosis destroy hilar vascularity, resulting in peripheral blood supply from inflamed perinodal soft tissue [26]. In our study, 33.3% of the CTBL cases showed peripheral vascularity patterns. These patterns may play an important role in identifying tuberculosis and other inflammatory LNs. According to the majority of published papers, other inflammatory LNs are characterized by a centrifugal and homogeneous enhancement pattern [7].

The principles of TB treatment for children are the same as adults, and anti-bacillary chemotherapy remains the main treatment for CTBL. Usually, a 6-month treatment course is considered adequate if the bacilli are susceptible to first-line drugs [4, 27]. Moreover, 20% of cases may show paradoxical upgrading reaction (PUR), which is defined as new or enlarging LNs, cold abscesses, a new sinus, etc., and the risk of PUR is higher in adolescents [6, 27]. Steroids have been considered as a means to reduce the robust immune response in PUR, but steroid use is controversial. Surgical excision was recommended only in unusual circumstances, such as significant and persistent nodal discomfort, common and uncomfortable PUR, prolonged antibiotic therapy and/or corticosteroids and disease cause by drug-resistant organisms [15].

The limitations of the present study included the lack of other imaging, such as CT. Owing to the high level of radiation to which the patient is exposed, only a few patients who were enrolled in the present study received CT. Second, the limitations also included the absence of images in children under one year of age. In addition, the infant and preschoolers clinical manifestations may be derived from the parents' chief complaints. Finally, because our hospital is a diagnosis and treatment center of tuberculosis, there were few metastatic and lymphoma lymph nodes cases in our samples. We will accumulate these cases in subsequent studies.

In summary, the ultrasonic signs of absent hilus, S/L ratio ≥ 0.5, unclear edge, necrosis, echogenic thin layer, strong echoes and capsular or peripheral vascularity were highly suggestive of CTBL in children from different age groups with night sweats, fatigue, low fever and other symptoms or seemingly healthy. Therefore, ultrasound could provide valuable information for the diagnosis of children with CTBL.
